# Associations Between Thyroid Volume and Physical Growth in Pubertal Girls: Thyroid Volume Indexes Need to Be Applied to Thyroid Volume Assessments

**DOI:** 10.3389/fendo.2021.662543

**Published:** 2021-05-19

**Authors:** Yingying Wang, Xiaolian Dong, Chaowei Fu, Meifang Su, Feng Jiang, Dongli Xu, Rui Li, Peixin Huang, Na Wang, Yue Chen, Qingwu Jiang

**Affiliations:** ^1^Department of Epidemiology, School of Public Health, Fudan University, Shanghai, China; ^2^Key Laboratory of Public Health Safety of Ministry of Education, School of Public Health, Fudan University, Shanghai, China; ^3^Department of Chronic Disease Control and Prevention, Deqing County Center for Disease Control and Prevention, Huzhou, China; ^4^Department of Chronic Disease Control and Prevention, Yuhuan City Center for Disease Control and Prevention, Taizhou, China; ^5^Department of Chronic Disease Control and Prevention, Minhang District Center for Disease Control and Prevention, Shanghai, China; ^6^Department of Chronic Disease Control and Prevention, Haimen City Center for Disease Control and Prevention, Nantong, China; ^7^School of Epidemiology and Public Health, Faculty of Medicine, University of Ottawa, Ottawa, ON, Canada

**Keywords:** thyroid volume, thyroid volume index, anthropometric index, puberty, girls

## Abstract

**Background:**

Thyroid volume (Tvol) is associated with many factors, but the current reference values for Tvol in children with sufficient iodine intake are inappropriate and need to be updated. Moderate changes in thyroid morphology and accentuated increases in body fat percentage occur during puberty as an adaption of the body and sexual development occurs. This study aimed to evaluate the influences of physical growth on Tvol and propose an easily applicable method for conducting Tvol assessments in pubertal girls with sufficient iodine intake.

**Methods**

A cohort study was conducted on 481 pubertal girls in East China from 2017 to 2019. B-ultrasound was used to assess Tvol. Multiple linear regression models were used to estimate the associations of Tvol enlargement (*d*Tvol) with changes in height (*d*H), weight (*d*W), waist circumference (*d*W), body mass index (*d*BMI), and body surface area (*d*BSA). Thyroid volume indexes (TVIs), including height thyroid volume index (HVI), weight and height thyroid volume index (WHVI), body mass index thyroid index (BMIV), and body surface area thyroid index (BSAV), were calculated to explore an appropriate method for Tvol assessments by Spearman correlation analyses.

**Results:**

Tvol, height, weight, BMI, and BSA increased significantly from baseline to follow-up (*P*<0.001). The associations between *d*Tvol and physical growth were only observed in the 13 to 14-year-old group. *d*H, *d*W,*d*BMI, and *d*BSA were positively related to *d*Tvol, with the maximum β of 5.74 (95%CI: 2.54 to 8.94) on *d*BSA, while *d*WC was negatively related to *d*Tvol (β= -0.05, 95%CI: -0.08 to -0.03). Both *d*HVI and *d*BSAV were not associated with *d*H, *d*W, *d*BMI, or *d*BSA in both age groups (*P*>0.05).

**Conclusions:**

Thyroid volume was associated with physical growth in pubertal girls in East China, both age and anthropometric measurements must be comprehensively considered to establish the reference values for Tvol. HVI, and BSAV may be better indicators for Tvol assessments in pubertal girls.

## Introduction

Goiter is considered one of the most common thyroid disorders in children and adolescents (1). Childhood goiter is linked to increased risks for thyroid cancer in adulthood (2), and the association may be more pronounced in female patients, due to the critical role of estrogen in thyroid tissues (3). Estrogen increases many-fold across pubertal development and stimulates thyroid cell proliferation and growth by binding to estrogen receptor (ER) (4). Puberty is initiated in late childhood through a cascade of neuroendocrine changes, resulting in extensive physical growth, sexual maturation, and surged estrogen levels in girls (5). Generally, moderate changes in thyroid morphology and accentuated increases in body fat percentage occur during puberty as an adaption to the body and sexual development (6).

Thyroid volume (Tvol) determined by B-ultrasound is strongly related to age, gender, puberty, body mass index (BMI), body surface index (BSA), and iodine nutrition status (7–9). Tvol also tends to increase with age in childhood (10). However, there exist many controversial issues in defining the reference values for Tvol in children, several studies have reported that the goiter rates were widely divergent when assessed both by using the regional reference values and the World Health Organization (WHO) reference values (11, 12), which were established in 2004 based on 3529 children living in areas with long-standing iodine sufficiency after adjustment for age and BSA (13). In addition, young people experience an accelerating secular trend for physical growth over two decades, and the pattern of physical growth is different between boys and girls (14). In addition, the current Chinese official reference values for Tvol in children are inappropriate. They were updated in 2007 but only considered age, ignoring physical growth factors (15) Therefore, more reasonable reference values for Tvol in children are warranted.

Previous studies have reported effective applications for assessing four types of thyroid volume index, including height volume index (HVI), weight-and-height volume index (WHVI), body mass volume index (BMIV), and body surface area volume index (BSAV) (16, 17). Based on a cohort in East China, this study aimed to estimate the correlations between Tvol and physical growth in pubertal girls with sufficient iodine intake, and propose an effective method for applying Tvol assessments in childhood for future study.

## Materials and Methods

### Study Design and Subjects

**Figure 1:**
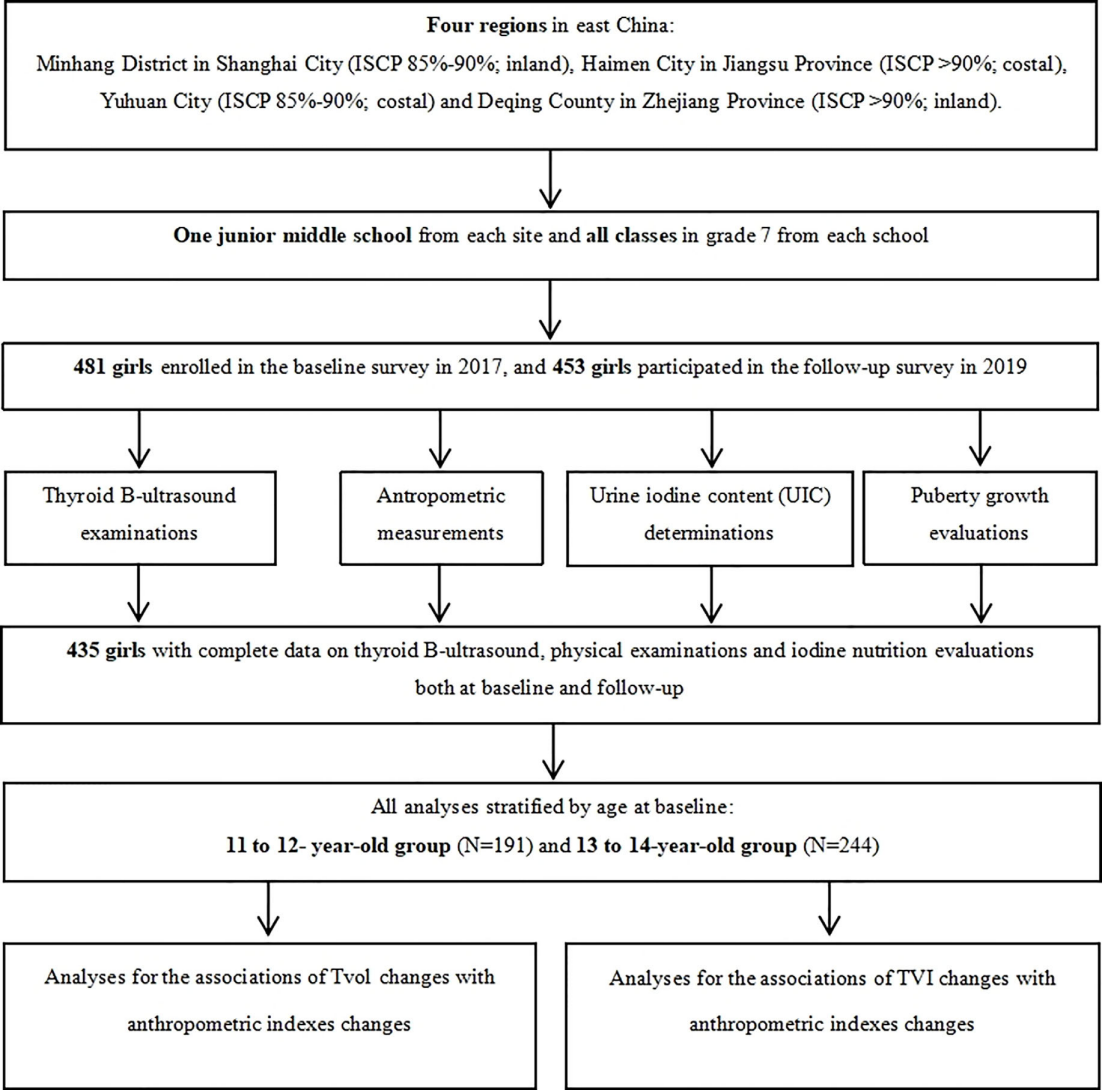
A flow chart for the study.

Four regions in East China (Minhang District in Shanghai City, Haimen City in Jiangsu Province, and Yuhuan City and Deqing County both in Zhejiang Province) were selected by a purposive sampling method. These four regions have different family iodized-salt consumption proportions (ISCP, 85%-90% for Minhang and Yuhuan, >90% for Haimen and Deqing). A detailed description of the study design and sampling method has been reported previously (18). A total of 481 girls in Grade 7 from a junior middle school from each site, who were mainly local residents, were selected in the cohort after excluding those with any previous history of iodine supplement consumption, thyroid disorders, or pituitary abnormalities. The baseline and follow-up investigation were conducted between October and November in 2017 and 2019, respectively, with the consistent investigation content. Ultimately, 435 girls had completed information on thyroid B-ultrasound and physical examinations. The response rate was 91.27%. A flow chart is presented in **Figure 1**.

### Thyroid B-Ultrasound Examinations

B-ultrasound has been an established tool for Tvol assessments (19). All participants, sitting upright with the neck completely extended, were received thyroid B-ultrasound examinations using a real-time sector scanner with a 7.5 MHz/40mm probe linear transducer and a standard technique (20). For each thyroid lobe, the maximal width (w, mm) was determined in the transverse section, and the maximal length (l, mm) and depth (d, mm) were measured in the longitudinal section, recorded to the nearest 0.01mm. Tvol was the sum of the volume of two lobes and calculated according to the function: V_L/R_(ml)= 0.479×l×w×t÷1000, V(ml)= V_L_+V_R_ (21).

### Anthropometric Measurements

Measurements for standing height (H, cm), weight (W, kg), and waist circumferences (WC, cm) were performed by trained health professionals using uniform anthropometric instruments. Height and WC were recorded to the nearest 0.1 cm, as well as weight was recorded to the nearest 0.1 kg. Body mass index (BMI) was calculated as weight in kilograms divided by the square of height in meters: BMI [kg/m (2)] =W÷(H÷100) (2); Body surface area (BSA) was calculated by the following formula: BSA (m^2^) =0.0061×H+0.0128×W-0.1529 (H: height, cm; W: weight, cm).

### Thyroid Volume Index Calculations

Four thyroid volume indexes, including height volume index (HVI), weight and height volume index (WHVI), BMI volume index (BMIV) and BSA volume index (BSAV) were calculated as follows: (1) HVI = V÷H×100; (2) WHVI = V÷ (W×H)×1000; (3) BMIV = V÷BMI×10; (4) BSAV = V÷BSA, V: thyroid volume, ml; H: height, cm; W: weight, kg; BMI: body mass index. kg/m^2^; BSA: body surface area, m^2^ (17).

### Urine Iodine Content (UIC) Determinations

Casual morning urine samples on a Monday and a Thursday (as the representatives for home diets and school canteen diets, respectively), approximately 10 to 12 ml, were obtained from all participants at each survey and then kept frozen at -80°C. The collected samples were determined for urine iodine content (UIC) by the method of inductively coupled plasma mass spectrometry (ICP-MS), and urine creatinine concentrations (UCr) by colorimetric enzymatic assay. Daily urine iodine output (DUIO) and weighted daily urine iodine output (WDUIO) were calculated to evaluate iodine nutrition: (1) daily urine iodine output: DUIO (μg) = MCr×1000×UIC÷UCr (18), MCr: daily urine creatinine output, mmol/day, MCr = EXP(0.0102×H-0.6854) (22); H: height, cm; UIC: urine iodine concentration, µg/L; UCr: urine creatinine content, µmol/L); (2) weighted daily urine iodine output: WDUIO (μg) =2/7× DUIO on Monday + 5/7× DUIO on Thursday (18).

### Puberty Growth Evaluations

The Puberty development scale (PDS) was used to evaluate the puberty development at each investigation. Puberty category scores (PCS) were calculated according to the total scores of three items including menarche (score 0 or 1, namely “without or with”), breast development (score 1 to 5), and body hair growth (score 1 to 4) (23).

### Statistical Analysis

Data on 435 girls with completed information of thyroid B-ultrasound and physical examinations were used for statistical analyses. Participants in our study were divided into two groups according to baseline age: Group 1, 11 to 12-year-old, and Group 2, 13 to 14-year-old.

Changes in each indicator were calculated using the following formula: *d*X = X_Follow-up_ - X_Baseline_, X refers to Tvol, H, W, WC, BMI, BSA, HVI, WHVI, BMIV, BSAV, PCS, or WDUIO, respectively. Wilcoxon matched-pairs signed-ranks test and paired t-test were used to compare Tvol, urine iodine output, and anthropometric indexes, respectively, between baseline and follow-up.

Multiple linear regression analyses were used to estimate the associations between Tvol changes (*d*Tvol) and changes in the anthropometric indexes (*d*H, *d*W, *d*WC, *d*BMI, and *d*BSA) after adjustment for puberty development and urine iodine levels.

Spearman correlation analyses were used to evaluate the associations between changes in the thyroid volume indexes (*d*HVI, *d*WHVI, *d*BMIV, and *d*BSAV) and changes in the anthropometric indexes (*d*H, *d*W, *d*WC, *d*BMI, and *d*BSA).

The level of statistical significance was defined as α = 0.05 of two-side probability. All analyses were performed using R program (version 4.0.4, R Foundation for Statistical Computing, Vienna, Austria), and all figures were performed by using GraphPad Prism software (version 7, GraphPad Prism, California, USA).

## Results

### Distributions of Tvol and Anthropometric Indexes

**Figure 2:**
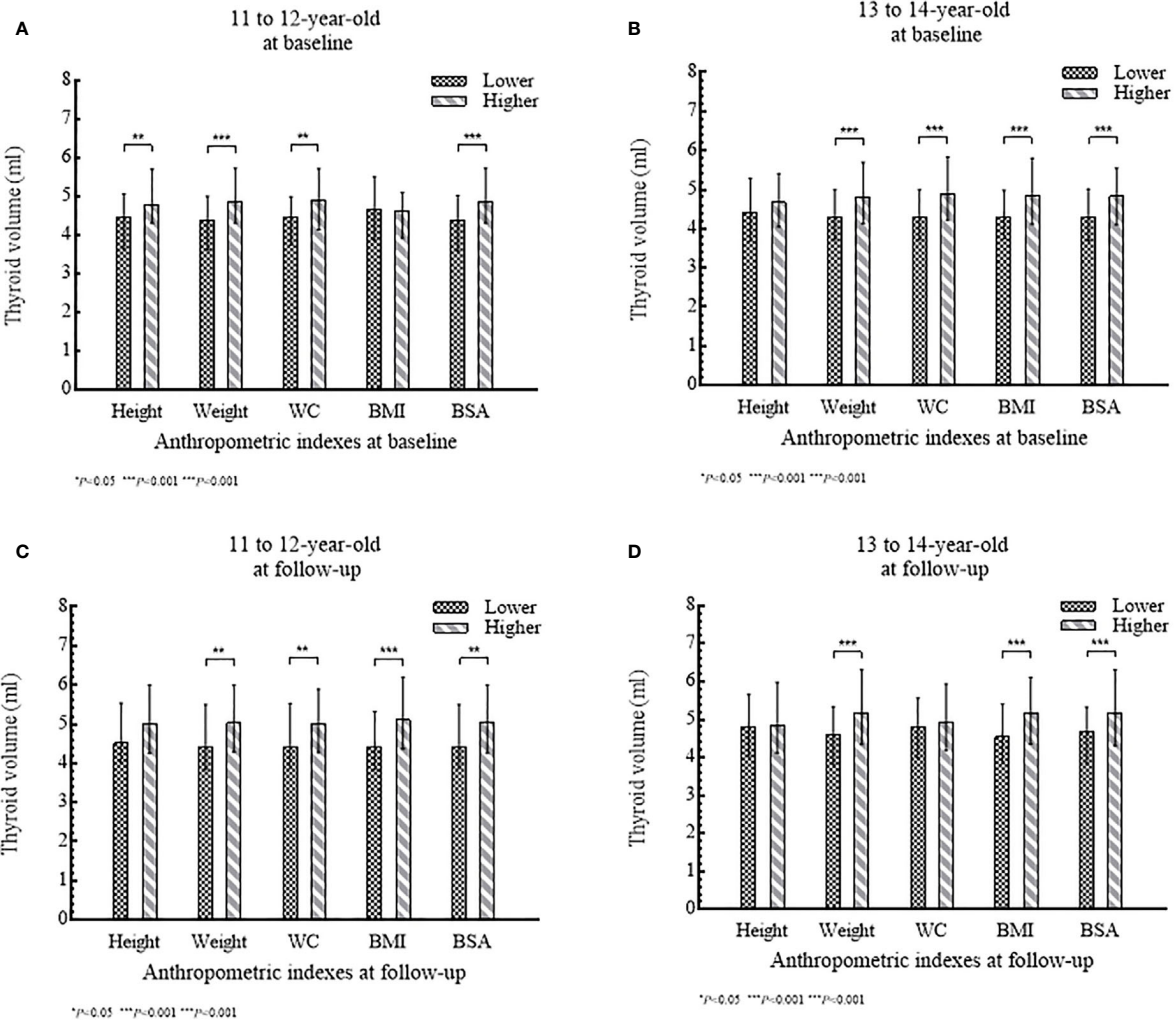
**(A–D)** Tvol according to anthropometric index (lower level or higher level) stratified by age group: **(A)** and **(B)** at baseline; **(C)** and **(D)** at follow-up.

Of 435 girls, Tvol, height, weight, BMI, BSA, and PCS increased significantly from baseline to follow-up in both age groups (*P*<0.001). The 13 to 14-year-old group had a slightly higher *d*Tvol, but it was not significant (*P*>0.05), compared to the 11 to 12-year-old group. Changes in anthropometric indexes except BMI were higher in the 11 to 12-year-old group than that in the 13 to 14-year-old group (*P*<0.001) (**Table 1**). The associations between Tvol and anthropometric indexes for the two investigations are shown in **Figure 2**.

**Table 1:** Distributions of Tvol, anthropometric indexes and other relative variables among 435 girls.

Age group	Characteristics	Baseline	Follow-up	Changes
**11 to 12-year-old (N=191)**	thyroid volume (ml)^a^	4.67(3.91~5.27)	4.67(4.10~5.55)^**^c^^	0.18(-0.51~0.94)
	height (cm)^b^	153.79 ± 6.42	159.50 ± 5.48^***^c^^	5.72 ± 3.48
	weight (kg)^b^	43.81 ± 9.30	50.26 ± 8.78^***^c^^	6.45 ± 5.45
	waist (cm)^b^	63.74 ± 7.27	68.93 ± 7.19^***^c^^	5.15 ± 5.74
	BMI (m ^2^/kg)^b^	18.43 ± 3.27	19.73 ± 3.10^***^c^^	1.29 ± 2.01
	BSA (m ^2^)^b^	1.38 ± 0.18	1.46 ± 0.13^***^c^^	0.08 ± 0.13
	WDUIO (μg)^a^	95.14(69.78~156.74)	96.07(78.07~125.00)	6.49(-40.28~35.9)
	PCS^b^	5.19 ± 1.67	7.36 ± 1.08^***^c^^	2.17 ± 1.32
**13 to 14-year-old (N=244)**	thyroid volume (ml)^a^	4.53(3.88~5.34)	4.78(4.09~5.74)^***^c^^	0.60(-0.55~1.25)
	height (cm)^b^	156.47 ± 5.54^***^d^^	159.29 ± 5.25^***^c^^	2.82 ± 2.56^***^d^^
	weight (kg)^b^	46.58 ± 8.15^**^d^^	51.49 ± 8.02^***^c^^	4.91 ± 3.58^***^d^^
	waist (cm)^b^	66.46 ± 7.90^***^d^^	67.23 ± 7.08^*^d^^	0.77 ± 6.15^***^d^^
	BMI (m ^2^/kg)^b^	18.99 ± 2.96	20.3 ± 3.04^***^c^^	1.30 ± 1.32
	BSA (m ^2^)^b^	1.43 ± 0.13^**^d^^	1.48 ± 0.12^***^c^^	0.05 ± 0.06^***^d^^
	WDUIO (μg)^a^	82.32(51.22~122.34)^***^d^^	83.98(71.25~103.67)^***^d^^	9.19(-27.28~35.08)
	PCS^b^	6.42 ± 1.34^***^d^^	7.92 ± 0.89^*** c ***^d^^	1.50 ± 1.14^***^d^^

a Data were presented as Median(P_25_~P_75_);

b Data were presented as Mean ± SD;

c Significantly different compared to baseline;

d Significantly different compared to the 11 to 12-year-old group.

^*^P<0.05; ^**^P<0.01; ^***^P<0.001.

### Associations of Tvol Changes (*d*Tvol) With Anthropometric Indexes Changes (*d*H, *d*W, *d*WC, *d*BMI, and *d*BSA)

After being adjusted for age, changes in puberty development, and urine iodine levels, the results of multiple linear regression analyses suggested that the influence of anthropometric indexes changes (*d*H, *d*W, *d*WC, *d*BMI, and *d*BSA) on *d*Tvol were observed in girls in the 13 to 14-year-old group. Increased height, weight, BMI, and BSA may stimulate Tvol enlargement, with the maximum β of 5.74 (95%CI:2.54 to 8.94) on *d*BSA in Model 2. However, *d*WC was negatively associated with *d*Tvol (β= -0.05, 95%CI: -0.08 to -0.03).

Further stratified analyses were conducted in girls with menarche or not at baseline, as well as those from area with different proportions of iodized-salt consumption. The positive associations between *d*Tvol and *d*H or *d*BSA were found in the 11 to 12-year-old group from Haimen or Deqing (**Table 2**). Among the 13 to 14-year-old group, compared with girls who experienced menarche at baseline, *d*Tvol in girls without menarche were more likely to be affected by changes in anthropometric indexes, with the maximum β of 9.78 (95%CI: 4.41 to 15.14) on *d*BSA in Model 2 (**Table 3**).

**Table 2:** Regression coefficients (βs) and 95% confidence intervals (95% CIs) for Tvol changes with anthropometric indexes changes by multiple linear regression models in 11 to 12-year-old group.

Anthropometricindexes changes	N	*d*Tvol^a^	β(95%CI)	
			Model 1^b^	Model 2^c^
***d*H #** PCS				
All	191	0.18(-0.51~0.94)	0.04(-0.02,0.1)	0.04(-0.01,0.1)
Area				
Minhang & Yuhuan	142	0.13(-0.54~0.88)	-0.04(-0.14,0.07)	-0.04(-0.14,0.07)
Haimen & Deqing	49	0.25(0.02~0.94)	0.06(0,0.13)	0.07(0.01,0.14)*
Menarche at baseline				
No	103	0.33(-0.38~1.03)	0.03(-0.05,0.12)	0.04(-0.05,0.12)
Yes	88	0.03(-0.55~0.68)	0.11(-0.01,0.23)	0.12(-0.01,0.24)
***d*W #** PCS				
All	191	0.18(-0.51~0.94)	0.02(-0.01,0.05)	0.03(-0.01,0.06)
Area				
Minhang & Yuhuan	142	0.13(-0.54~0.88)	0(-0.08,0.07)	0(-0.09,0.08)
Haimen & Deqing	49	0.25(0.02~0.94)	0.02(-0.01,0.06)	0.03(-0.01,0.07)
Menarche at baseline				
No	103	0.33(-0.38~1.03)	0.03(-0.04,0.1)	0.05(-0.04,0.13)
Yes	92	0.03(-0.55~0.68)	0.02(-0.02,0.05)	0.02(-0.01,0.05)
***d*WC** $				
All	191	0.18(-0.51~0.94)	-0.01(-0.04,0.02)	-0.01(-0.04,0.02)
Area				
Minhang & Yuhuan	142	0.13(-0.54~0.88)	0(-0.06,0.05)	0(-0.06,0.05)
Haimen & Deqing	49	0.25(0.02~0.94)	-0.02(-0.06,0.02)	-0.03(-0.07,0)
Menarche at baseline				
No	103	0.33(-0.38~1.03)	-0.01(-0.06,0.04)	-0.01(-0.06,0.04)
Yes	88	0.03(-0.55~0.68)	-0.02(-0.06,0.02)	-0.03(-0.06,0.01)
***d*BMI #**				
All	191	0.18(-0.51~0.94)	0.04(-0.05,0.12)	0.05(-0.04,0.14)
Area				
Minhang & Yuhuan	142	0.13(-0.54~0.88)	0.02(-0.19,0.23)	0.03(-0.2,0.27)
Haimen & Deqing	49	0.25(0.02~0.94)	0.04(-0.06,0.13)	0.05(-0.04,0.15)
Menarche at baseline				
No	103	0.33(-0.38~1.03)	0.06(-0.14,0.26)	0.1(-0.13,0.32)
Yes	88	0.03(-0.55~0.68)	0.02(-0.06,0.11)	0.04(-0.05,0.12)
***d*BSA #**				
All	191	0.18(-0.51~0.94)	0.06(-1.59,1.71)	0.16(-1.51,1.83)
Area				
Minhang & Yuhuan	142	0.13(-0.54~0.88)	-0.75(-3.54,2.05)	-0.74(-3.57,2.09)
Haimen & Deqing	49	0.25(0.02~0.94)	2.18(-0.39,4.75)	2.82(0.16,5.48)*
Menarche at baseline				
No	103	0.33(-0.38~1.03)	0.12(-2.91,3.14)	0.28(-2.85,3.41)
Yes	88	0.03(-0.55~0.68)	1.72(-0.86,4.3)	2.19(-0.47,4.85)

a The median and quartiles [Median(P_25_~P_75_)] of Tvol changes;

b Model 1: Adjusted for age at baseline, puberty category scores changes(dPCS) and weighted daily urine iodine output changes (dWDUIO);

c Model 2: Based on Model 1, # additionally adjusted for dWC; $ additionally adjusted for dBSA.

^*^P<0.05.

**Table 3:** Regression coefficients (βs) and 95% confidence intervals (95% CIs) for Tvol changes with anthropometric indexes changes by multiple linear regression models in 13 to 14-year-old group.

Anthropometricindexes changes	N(%)	*d*Tvol^a^	β(95%CI)	
			Model 1^b^	Model 2^c^
***d*H #** PCS				
All	244	0.60(-0.55~1.25)	0.1(0.03,0.18)**	0.1(0.02,0.17)**
Area				
Minhang & Yuhuan	78	-0.31(-0.9~0.33)	0.11(-0.03,0.25)	0.1(-0.04,0.25)
Haimen & Deqing	166	0.61(-0.01~1.28)	0.11(0.02,0.19)*	0.1(0.01,0.18)*
Menarche at baseline				
No	57	0.32(-0.5~0.98)	0.19(0.07,0.31)**	0.18(0.06,0.3)**
Yes	187	0.34(-0.4~1.07)	0.12(0.02,0.22)*	0.1(0,0.2)*
***d*W #** PCS				
All	244	0.18(-0.51~0.94)	0.05(0,0.09)*	0.08(0.03,0.13)***
Area				
Minhang & Yuhuan	78	0.13(-0.54~0.88)	0.09(0.02,0.16)*	0.13(0.05,0.21)**
Haimen & Deqing	166	0.25(0.02~0.94)	0.01(-0.05,0.07)	0.05(-0.02,0.11)
Menarche at baseline				
No	57	0.33(-0.38~1.03)	0.09(0,0.18)*	0.16(0.06,0.25)**
Yes	187	0.03(-0.55~0.68)	0.04(-0.02,0.09)	0.07(0.01,0.13)*
***d*WC** $				
All	244	0.18(-0.51~0.94)	-0.04(-0.06,-0.01)**	-0.05(-0.08,-0.03)***
Area				
Minhang & Yuhuan	78	0.13(-0.54~0.88)	-0.02(-0.07,0.04)	-0.05(-0.11,0)
Haimen & Deqing	166	0.25(0.02~0.94)	-0.05(-0.08,-0.02)**	-0.06(-0.09,-0.03)***
Menarche at baseline				
No	57	0.33(-0.38~1.03)	-0.04(-0.09,0.01)	-0.07(-0.12,-0.02)**
Yes	187	0.03(-0.55~0.68)	-0.04(-0.07,-0.01)*	-0.05(-0.08,-0.02)**
***d*BMI #**				
All	244	0.18(-0.51~0.94)	0.06(-0.07,0.18)	0.17(0.03,0.3)*
Area				
Minhang & Yuhuan	78	0.13(-0.54~0.88)	0.18(-0.01,0.37)	0.28(0.06,0.49)*
Haimen & Deqing	166	0.25(0.02~0.94)	-0.05(-0.02,0.11)	0.07(-0.11,0.25)
Menarche at baseline				
No	57	0.33(-0.38~1.03)	0.13(-0.11,0.38)	0.36(0.08,0.64)*
Yes	187	0.03(-0.55~0.68)	0.03(-0.11,0.17)	0.13(-0.03,0.28)
***d*BSA #**				
All	244	0.18(-0.51~0.94)	3.84(0.7,6.97)*	5.74(2.54,8.94)***
Area				
Minhang & Yuhuan	78	0.13(-0.54~0.88)	6.88(1.62,12.14)*	8.99(3.31,14.67)**
Haimen & Deqing	166	0.25(0.02~0.94)	1.9(-2.06,5.85)	3.94(-0.02,7.89)
Menarche at baseline				
No	57	0.33(-0.38~1.03)	7.54(2.15,12.93)**	9.78(4.41,15.14)***
Yes	187	0.03(-0.55~0.68)	2.99(-0.88,6.87)	4.89(0.92,8.86)*

a The median and quartiles [Median(P_25_~P_75_)] of Tvol changes;

b Model 1: Adjusted for age at baseline, puberty category scores changes(dPCS) and weighted daily urine iodine output changes (dWDUIO);

c Model 2: Based on Model 1, # additionally adjusted for dWC; $ additionally adjusted for dBSA.

^*^P<0.05; ^**^P<0.01; ^***^P<0.001.

### Associations of Thyroid Volume Indexes Changes (*d*HVI, *d*WHVI, *d*BMIV, and *d*BSAV) With Anthropometric Indexes Changes (*d*H, *d*W, *d*WC, *d*BMI, and *d*BSA)

**Figure 3:**
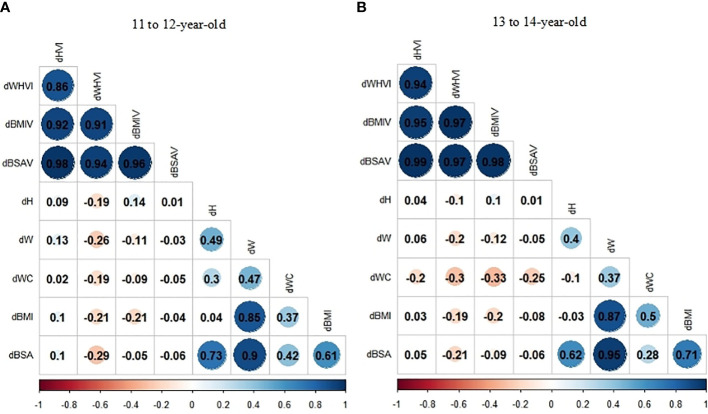
**(A, B)** Correlation coefficients (ρs) of the relationship between thyroid volume indexes (TVI) changes and anthropometric indexes changes stratified by age group by Spearman correlation analyses: **(A)**11 to 12-year-old group; **(B)** 13 to 14-year-old group.

The heatmap of spearman correlation analyses indicates the independence for *d*HVI and *d*BSAV (**Figure 3**). Among the 11- to 12-year-old group, neither *d*HVI nor *d*BSAV were associated with all anthropometric indexes (*d*H, *d*W, *d*WC, *d*BMI, and *d*BSA) (*P*>0.05), with the modulus of correlation coefficients (ρs) less than 0.10. Among the 13- to 14-year-old group, neither *d*HVI nor *d*BSAV were associated with *d*H, *d*W, *d*BMI, and *d*BSA (*P*>0.05). On the contrary, *d*WHVI was negatively related to all anthropometric indexes, with the maximum ρ modulus of -0.30 with *d*WC in the 13- to 14-year-old group.

Therefore, HVI and BSAV may be better indicators for establishing reference values for Tvol, with fewer associations with physical growth. The percentiles for HVI and BSAV by age are presented in **Table 4**.

**Table 4:** Percentile values for height thyroid volume index (HVI) and body surface area thyroid volume index (BSAV) stratified by age in girls aged 11 to 16 years.

Age (years)^a^	N^b^	HVI							BSAV						
		*P_5_*	*P_10_*	*P_25_*	*P_50_*	*P_75_*	*P_90_*	*P_95_*	*P_5_*	*P_10_*	*P_25_*	*P_50_*	*P_75_*	*P_90_*	*P_95_*
**11**	39	2.17	2.30	2.54	3.00	3.47	4.06	4.41	2.45	2.66	2.95	3.43	4.00	4.36	5.20
**12**	156	2.07	2.27	2.60	2.97	3.51	4.20	4.76	2.38	2.53	2.89	3.23	3.93	4.62	5.09
**13**	272	2.13	2.26	2.52	2.90	3.38	4.11	4.53	2.34	2.49	2.80	3.23	3.74	4.40	4.95
**14**	163	1.94	2.16	2.54	2.95	3.67	4.29	5.32	2.17	2.47	2.74	3.20	3.92	4.82	5.40
**15**	234	2.11	2.30	2.57	3.07	3.65	4.33	4.82	2.38	2.52	2.78	3.31	3.89	4.69	5.16
**16**	10	2.12	2.13	2.23	2.57	4.09	4.93	-^c^	1.98	2.02	2.46	2.92	4.28	5.08	-^c^

a Age at investigation;

b Numbers of records in each age;

c Lacking due to deficient sample size.

## Discussions

Tvol is influenced by iodine nutrition status (24) and non-iodine factors (25). In this longitudinal cohort study, given the appropriate proportions of iodized-salt consumption in all sites, and the iodine-sufficient status for the target population, the effects of iodine-deficiency on Tvol are lesser discussed.

In our study, an increasing trend for Tvol, height, weight, WC, BMI, and BSA were observed during the study progress. We found that changes in height, weight, BMI, and BSA were positively related to Tvol enlargement, which was consistent with the results in children aged 8 to 10 years from East China in previous studies (16, 17). Similarly, positive correlations between Tvol and height or weight were also reported in Brazilian children aged 6 to 14 years by Svensson et al. (26), and in Swedish children aged 7 to 18 years by Lisboa (27). BSA was calculated according to height and weight and considered as a key predictor for Tvol, followed by age (8, 28). BMI can be seen as the adjusted-weight by height and the most frequently-used indicator to evaluate obesity status (29), larger Tvol was observed in the BMI-based obese children by Soydan et al. (30) Accentuated increases in body fat percentage occurs after the onset of puberty, when sex differences in overall and regional body composition become more visible, and girls may have more than twice the fat mass index (FMI) of boys (31). Adipocytes secrete a larger number of inflammatory cytokines, impairing sodium/iodine symporter function and accelerating thyrocyte proliferation (32). Another hypothesis is that leptin, a hormone produced by adipocytes, stimulates TSH secretion by hypothalamic-pituitary axis. TSH can stimulate the growth and metabolic activity of thyroid follicular cells (33). Therefore, more attention should be paid to body composition changes and body fat accumulation in pubertal girls to promptly recognize and reduce the risk for abnormal thyroid enlargement.

However, we found that *d*WC was negatively associated with *d*Tvol. An appropriate interpretation of the results is that compared to other anthropometric indexes, which are based on height or weight, WC may represent physical growth in the horizontal dimension. In addition, the influences of anthropometric characteristics on Tvol in our study were established only in the 13 to 14-year-old group but not in the 11 to 12-year-old group. The landmarks of the pubertal events in girls are peak height velocity (PHV) and menarche. Menarche is a rather late event in puberty and usually occurs 6 months after PHV (34), which can be achieved at around 12 years old (35). Girls in the 11 to 12-year-old group were more likely to be in the early pubertal stage with less estrogen accumulation and lower baseline anthropometric characteristics values than the 13 to 14-year-old group. In stratified analyses, we further found the positive associations between *d*Tvol and *d*H or *d*BSA in the 11 to 12-year-old group living in Haimen or Deqing, the areas with >90% iodized-salt consumption proportion, but there was no interaction between anthropometric indexes and area factor.

Chen et al. found that the goiter rates were widely divergent when assessed by WHO criterion and by Chinese criterion (33% vs. 10.9%) (24), the current WHO reference values for Tvol in children were proposed in 2004 based on age and BSA (13), while the Chinese criterion were established in 2007 based on age (15), meaning that the reference intervals for Tvol need to be adjusted and updated. In our study, the appropriate age-specific thyroid volume indexes (TVIs) are proposed. Consistent with previous studies (36, 37), HVI and BSAV are well-applicable for Tvol assessments in pubertal girls.

There exists some limitations in our study. First, subjects were residing in the iodine-sufficient regions in East China, so the reference values established in our study may not be fully applicable to other populations, but the thyroid volume indexes, after adjusting for the influences of physical growth, are worthy of being recommended. Second, due to the non-substantial sample size, subjects were divided into different age stratification with an interval of every two years as opposed to one year. Third, we failed to observe evident associations between *d*Tvol and changes in the anthropometric indexes in the 11 to 12-year-old group, and we would expand the sample size for further observations and discussions in future studies. To our knowledge, this is one of few studies that explore the relationship between Tvol and physical growth in pubertal girls. It is important to consider youths in this age group who are in a critical growth period with increasing FMI and increasing Tvol, and the results from this study provide some evidence for goiter assessments in children during puberty.

## Conclusions

Thyroid volume is associated with physical growth in pubertal girls in East China, and both age and anthropometric measurements must be comprehensively considered to establish the reference values for Tvol. HVI and BSAV may be better indicators for Tvol assessments in pubertal girls.

## Data Availability Statement

The datasets for this article are not publicly available because it contains personal information of participants. Requests to access the datasets should be directed to NW, na.wang@fudan.edu.cn.

## Ethics Statement

The study was approved by the ethical review board of School of Public Health of Fudan University (#2012-03-0350S). Written informed consent to participate in this study was provided by the participants’ legal guardian/next of kin.

## Author Contributions

XD, MS, DX, JQ, NW, and QJ contributed to the study design. YW, CF, FJ, RL, and NW contributed to data acquisition and collection. YW, NW, and YC contributed to data analysis and interpretation. YW, NW, and YC drafted the manuscript. All authors contributed to the article and approved the submitted version.

## Funding

This work was supported by grants from the National Natural Science Foundation of China (Grant No. 81602806).

## Conflict of Interest

The authors declare that the research was conducted in the absence of any commercial or financial relationships that could be construed as a potential conflict of interest.

## Abbreviations

Tvol, thyroid volume; H, height; W, weight; WC, waist circumference; BMI, body mass index; BSA, body surface area; TVI, thyroid volume index; HVI, height volume index; WHVI, weight height volume index; BMIV, body mass volume index; BSAV, body surface area volume index; DUIO, daily urine iodine output; WDUIO, weighted daily urine iodine output; UIC, urine iodine content; UCr, urine creatinine content; PDS, puberty development scale; PCS, pubertal category scores; ISCP, iodized-salt consumption proportions; ICP-MS, inductively coupled plasma mass spectrometry; PHV, peak height velocity; FMI, fat mass index.
